# Oral cavity metastasis: An unusual presentation of carcinoma prostate

**DOI:** 10.4103/0970-1591.38615

**Published:** 2008

**Authors:** Dileep Damodaran, N. Kathiresan, B. Satheesan

**Affiliations:** Division of Genitourinary Oncology, Department of Surgical Oncology, Cancer Institute (W.I.A.), Sardar Patel Road, Adyar, Chennai, Tamil Nadu, India

**Keywords:** Oral cavity metastasis, prostate cancer, prostate specific antigen immunohistochemistry

## Abstract

Oral cavity cancers form the third most common cancers among men in south India. The oral cavity is a very rare site for metastases and has been described in various cancers, particularly lung, breast, kidney and colon carcinoma. Here a very rare case of a buccal metastasis from prostate carcinoma that was originally evaluated as a primary oral cavity malignancy is presented. Histopathological examination of a biopsy of the lesion revealed papillary adenocarcinoma Grade II, nuclear Grade II, which initiated the evaluation of prostate. On evaluation diagnosis of carcinoma prostate was made which was confirmed by immunohistochemistry for PSA.

## INTRODUCTION

Prostate cancer most commonly metastasizes to bone and lymph nodes, however, several metastatic sites remain covert and are rarely discovered ante mortem. These uncommon sites may be unfamiliar not only to most pathologists, but also to treating physicians. In south India oral cavity cancers form the third most common cancer among males.[1] We report a male patient who presented to our center as a primary oral cavity cancer which on evaluation was found to be a metastasis from advanced prostatic adenocarcinoma. Metastasis to gingivum from prostatic adenocarcinoma had been reported in the literature.[2] But buccal mucosal metastasis for any primary is very rare.[3] A brief review of the literature regarding advanced cancer prostate with unusual presentations is reviewed and discussed herein.

## CASE REPORT

**Figure 1:**
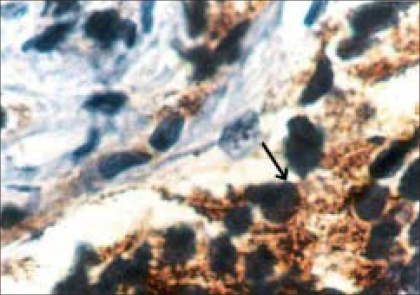
Black arrow showing tumor cells with cytoplasmic PSA positivity (× 100)

A 68-year-old elderly gentleman with trismus and painful ulcer right buccal mucosa presented to us in poor performance status (Eastern Cooperative Oncology Group (ECOG) performance status -3). Clinically he had moderate trismus and an ulceroproliferative growth of 4 × 3 cm involving the posterior buccal mucosa extending to the retro molar trigone on the right side. No significant cervical lymphadenopathy was noted. A wedge biopsy from the oral lesion was done, which revealed papillary adenocarcinoma Grade II, intermediate nuclear grade. The pathologist suggested the possibility of secondary deposit from prostate cancer and other glandular sites. This initiated the search for primary disease in other glandular sites. Subsequent examination revealed nodular prostatomeagly, which led to serum PSA and a tru-cut biopsy from the enlarged prostate. Serum PSA was elevated (41 ng/ml) and the histopathology of the tru-cut biopsy of the prostate revealed poorly differentiated carcinoma (Gleason score 4 + 3 combined Gleason 7). In order to confirm, immunohistochemistry studies for PSA on both the prostatic and buccal mucosal lesion were done. Both were found to be immuno-reactive [Figure 1].

The option of castration was offered, but the patient could not comply for the same due to logistics. He was put on antiandrogen (bicalutamide 150 mg once a day) and advised symptomatic and supportive care. He was lost to follow-up.

## DISCUSSION

Metastatic lesions to the oral cavity from distant tumors are uncommon, accounting for only 1% of all oral malignancies. They mainly involve the bony structures (particularly the mandible), whereas primary metastases to soft tissues are extraordinarily rare (only 0.1% of oral malignancies). The most common sites of soft tissue involvement are the gingiva, tongue, lips and the buccal and palatal mucosa. The primary tumors are mainly lung, breast, kidney and colon.[3]

Prostate cancer metastasizing to soft tissues in the oral cavity as a presenting feature has been anecdotally reported in the surgical literature.[2] But gingivum had been the site described – it may be the extension of bone metastasis involving the jaw bones. Diagnosis requires a high index of suspicion especially if the predominant presentation is oral cavity lesion. Immunohistochemistry with prostate-specific antigen (PSA) – and prostate-specific acid phosphatase (PSAP) would be helpful.

Prostate-specific antigen is a 30-kDa glycoprotein serine protease that shows high tissue specificity for prostatic tissue, both benign and malignant. However, recent reports have shown that a variety of normal and neoplastic tissue types express PSA immunohistochemically. One diagnostic pitfall is the localization of PSA-like immunoreactivity in human salivary glands. This is usually seen in the apical cytoplasm along the luminal border of small-sized duct epithelial cells of major salivary (parotid and submandibular) glands of both sexes.[4] However, in large-sized duct epithelial cells, acinar cells and minor salivary gland ducts it may be negative. Since our patient had a histologically proven prostate cancer, it suggests the oral lesion to be metastatic from the prostate.

Recently, studies have demonstrated that FDG-PET scanning may in fact play an important role in the evaluation of advanced androgen-independent disease and in staging and evaluation of response to hormonal manipulations in high-risk localized and locally advanced prostate cancers.[5] So in future FDG-PET may be helpful in detecting these rare metastatic sites ante mortem.

Metastastic prostate cancer carries poor prognosis and how the ante mortem diagnosis of these unusual metastatic sites dictates management and prognosis is largely unknown. Management strategies remain the same essentially. Castration, surgical or medical, is the standard of care. Hormonal manipulation does not always result in a favorable response. The degree of differentiation may be a good prognostic indicator in this regard. However, no large experience exists in the literature in treating such cases.

## CONCLUSION

Carcinoma of the prostate may rarely metastasize to soft tissues in the oral cavity; hence it is important to bear this possibility in mind because such conditions may mimic a different primary or a benign disease.
